# Regulation of Benzo[a]pyrene-Induced Hepatic Lipid Accumulation through CYP1B1-Induced mTOR-Mediated Lipophagy

**DOI:** 10.3390/ijms25021324

**Published:** 2024-01-22

**Authors:** Kyung-Bin Bu, Min Kim, Min Kyoung Shin, Seung-Ho Lee, Jung-Suk Sung

**Affiliations:** Department of Life Science, Dongguk University-Seoul, Goyang 10326, Republic of Korea; rudqls1211@dongguk.edu (K.-B.B.); pipikimmin@dongguk.edu (M.K.); shinmk94@dgu.ac.kr (M.K.S.); q969@dongguk.edu (S.-H.L.)

**Keywords:** non-alcoholic fatty liver disease, benzo[a]pyrene, CYP1B1, mTOR, lipophagy

## Abstract

Metabolic dysfunction-associated steatotic liver disease (MASLD) is caused by lipid accumulation within the liver. The pathogenesis underlying its development is poorly understood. Benzo[a]pyrene (B[a]P) is a polycyclic aromatic hydrocarbon and a group 1 carcinogen. The aryl hydrocarbon receptor activation by B[a]P induces cytochrome P450 (CYP) enzymes, contributing to hepatic lipid accumulation. However, the molecular mechanism through which the B[a]P-mediated induction of CYP enzymes causes hepatic lipid accumulation is unknown. This research was conducted to elucidate the role of CYP1B1 in regulating B[a]P-induced lipid accumulation within hepatocytes. B[a]P increased hepatic lipid accumulation, which was mitigated by CYP1B1 knockdown. An increase in the mammalian target of rapamycin (mTOR) by B[a]P was specifically reduced by CYP1B1 knockdown. The reduction of mTOR increased the expression of autophagic flux-related genes and promoted phagolysosome formation. Both the expression and translocation of TFE3, a central regulator of lipophagy, were induced, along with the expression of lipophagy-related genes. Conversely, enhanced mTOR activity reduced TFE3 expression and translocation, which reduced the expression of lipophagy-related genes, diminished phagolysosome production, and increased lipid accumulation. Our results indicate that B[a]P-induced hepatic lipid accumulation is caused by CYP1B1-induced mTOR and the reduction of lipophagy, thereby introducing novel targets and mechanisms to provide insights for understanding B[a]P-induced MASLD.

## 1. Introduction

Metabolic dysfunction-associated steatotic liver disease (MASLD) is characterized by lipid droplets (LDs) that accumulate in the liver [1,2]. MASLD is the name adopted in 2023 for the condition previously known as non-alcoholic fatty liver disease (NAFLD). Recent studies have shown that over 30% of the global population suffers from MASLD, and its prevalence is increasing as obesity rises worldwide [3,4,5]. It covers a wide range of disorders, from simple steatosis to non-alcoholic steatohepatitis [6]. It initiates as simple steatosis and often progresses to more severe stages, including liver fibrosis, cirrhosis, and even hepatocellular carcinoma [7,8,9]. Moreover, MASLD increases the risk of liver-related diseases, as well as the risk of various metabolic and cardiovascular disorders. While MASLD is currently recognized as a global public health problem, there is no well-established treatment. The most effective approach is in preventing MASLD progression by inhibiting intracellular lipid accumulation and inflammatory responses [3]. Given the lack of a comprehensive understanding of the underlying mechanisms, it is necessary to elucidate the molecular processes that influence the onset and progression of MASLD [10].

The development of MASLD is influenced by a combination of genetic predisposition and environmental factors, which are influenced by various other conditions, such as obesity, hypertension, type 2 diabetes, impaired lipid metabolism, and oxidative stress. Furthermore, exposure to endocrine disruptors (EDCs) is also known to contribute to the progression of MASLD [11,12]. Notably, benzo[a]pyrene (B[a]P) is a representative example of an EDC that can trigger MASLD [13,14]. B[a]P is a polycyclic aromatic hydrocarbon (PAH), which is classified as a first-class carcinogen by the International Agency for Research on Cancer (IARC) [15]. The body is exposed to this compound from air pollutants, such as cigarette smoke and engine exhaust fumes, in addition to smoked, barbecued, or overcooked meat products [16,17]. Moreover, environmental exposure to B[a]P can induce hepatic lipid accumulation, which can lead to hepatic steatosis and subsequent fibrosis [11,16,18]. However, the detailed mechanism through which B[a]P causes hepatic lipid accumulation is elusive and requires in-depth research.

B[a]P is known to be a direct ligand of the aryl hydrocarbon receptor (AhR). The activation of AhR by B[a]P regulates the expression of cytochrome P450 (CYP) enzymes, which are phase I enzymes in xenobiotic detoxification and include the CYP family 1 subfamily A member 1 (CYP1A1), CYP family 1 subfamily A member 2 (CYP1A2), and CYP family 1 subfamily B member 1 (CYP1B1) [19,20]. It is known that there are about 57 CYP enzyme genes in humans, of which CYP1A1 and CYP1B1 are representative [21,22,23,24]. These genes are involved in hormone synthesis and degradation, as well as xenobiotic metabolism and reactive oxygen species (ROS) production. In humans, the CYP enzymes drive nearly 70% of oxidative metabolism and approximately 50% of clinical drug detoxification and elimination [25,26]. Notably, these genes significantly influence the regulation of AhR-mediated lipid production, consequently contributing to the accumulation of lipids in the liver [27,28]. However, the precise mechanism for this process remains elusive. While some studies have demonstrated that the activation of AhR by B[a]P induces CYP1A1 to cause MASLD, additional research on CYP1B1 remains inadequate [16,28]. Therefore, the main objective of this study was to investigate the underlying mechanisms involved in the B[a]P-mediated induction of CYP1B1-driven lipid accumulation in hepatocytes.

Lipid metabolism in the liver, such as systemic lipid metabolism, can be broadly categorized into two fundamental processes: lipolysis and lipophagy. Lipolysis involves the metabolic breakdown of cytoplasmic LDs, facilitated by a cascade of lipases [29]. On the other hand, lipophagy is a mechanism that degrades intracellular LDs by activating autophagy-related pathways [30]. Specifically, lipophagy is a type of macroautophagy that engages double-membrane vesicles (autophagosomes) to sequester intracellular proteins or organelles, followed by the activation of lysosomes, and there is growing evidence indicating the dysregulation of lipophagy in MASLD. Lipophagy involves a precisely regulated multistep mechanism, including (1) the protein-mediated sequestration of LDs within a cytoplasmic double-membrane vesicle, resulting in phagosome formation; (2) tracking autophagosomes and transporting them to lysosomes; (3) the formation of autophagolysosomes through fusion between autophagosomes and lysosomes; and (4) cargo degradation by lysosomal lipases [31]. Currently, significant attention has been paid to elucidating the complex interplay between lipid metabolism and autophagy. Therefore, it has been identified that lipophagy can be a new target for MASLD [32,33,34].

The mammalian target of rapamycin (mTOR) is a major regulator of cell growth and development. It regulates gene transcription and nutrient uptake in response to intracellular nutrients, stressors, and energy levels, influencing cell growth, aging, and metabolism. Interestingly, it has been found that mTOR is also involved in lipid metabolism, where it has been discerned that it suppresses the induction of lipophagy by inhibiting autophagosome formation [35,36]. Moreover, mTOR regulates the expression of lysosome-related genes by controlling the translocation of the microphthalmia subfamily (MiTF/TFE family: MITF, TFEB, TFE3, and TFEC), which are known as master transcription factors of lipophagy [37,38]. Recently, progress has been made in elucidating the role of mTOR in lipid metabolism, thereby making it an attractive target for MASLD. Therefore, in this study, we focused on the correlation between mTOR and CYP1B1, along with their downstream genes, to elucidate the complex relationship between B[a]P-induced hepatic lipid accumulation and lipophagy.

## 2. Results

### 2.1. B[a]P-Induced Lipid Accumulation Is Decreased by CYP1B1 Knockdown in Hepatic Cells

**Figure 1:**
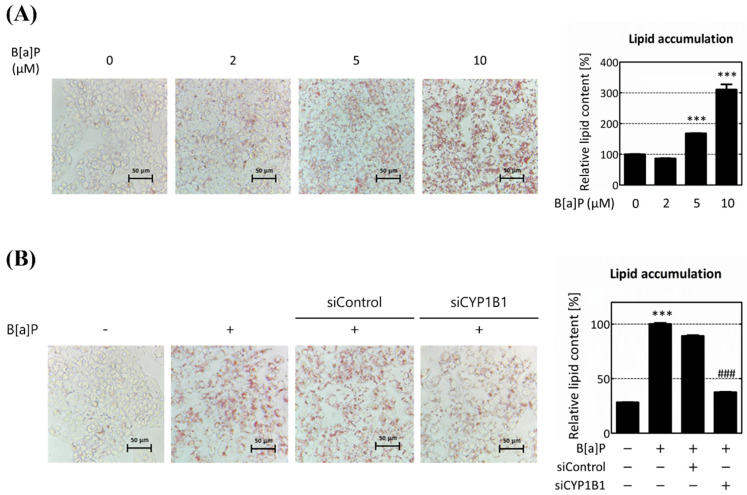
CYP1B1 knockdown reduces B[a]P-induced hepatic lipid accumulation. HepG2 cells were subjected to Oil-Red O (ORO) staining to assess lipid accumulation. (**A**) The quantification of lipid accumulation in hepatocytes following B[a]P treatment at concentrations ranging from 2 μM to 10 μM. (**B**) Lipid accumulation after CYP1B1 knockdown in B[a]P exposure conditions. *** *p* < 0.001, compared with the non-treated group; ### *p* < 0.001, compared with the siControl group. B[a]P: benzo[a]pyrene (10 μM). All experiments were performed in triplicate and repeated three times.

The B[a]P concentration used in this study was determined by evaluating the accumulation of intracellular lipids in hepatocytes following B[a]P treatment using various concentrations. After exposing the cells to a B[a]P concentration range of 2 μM to 10 μM for 48 h, we confirmed intracellular lipid accumulation using Oil-Red O (ORO) staining. At the lowest concentration of 2 μM, we observed an 86.54% of lipid accumulation compared to the non-treated group, although this result was not statistically significant. However, at concentrations of 5 μM and 10 μM, the accumulation of lipids notably increased to 168.24% and 310.57%, respectively (Figure 1A). These results indicate that B[a]P treatment increases intracellular lipid accumulation. The highest increase was noted at a treatment concentration of 10 μM, thereby suggesting that it is suitable for further investigation. To explore the relationship between CYP1B1 and B[a]P-induced hepatic lipid accumulation, we examined the lipid contents following CYP1B1 knockdown. In the B[a]P-treated group, lipid accumulation increased to approximately 354.53% compared to the non-treated group. Conversely, in the siControl group, lipid levels decreased to 89.01% compared to the B[a]P-treated group. Remarkably, in the CYP1B1 knockdown group, lipid accumulation dropped significantly to 37.51% compared to the B[a]P-treated group and 42.15% compared to the siControl group (Figure 1B). These results indicate that CYP1B1 promotes B[a]P-induced hepatic lipid accumulation.

### 2.2. CYP1B1 Specifically Regulates mTOR and Macroautophagy-Related Genes

**Figure 2:**
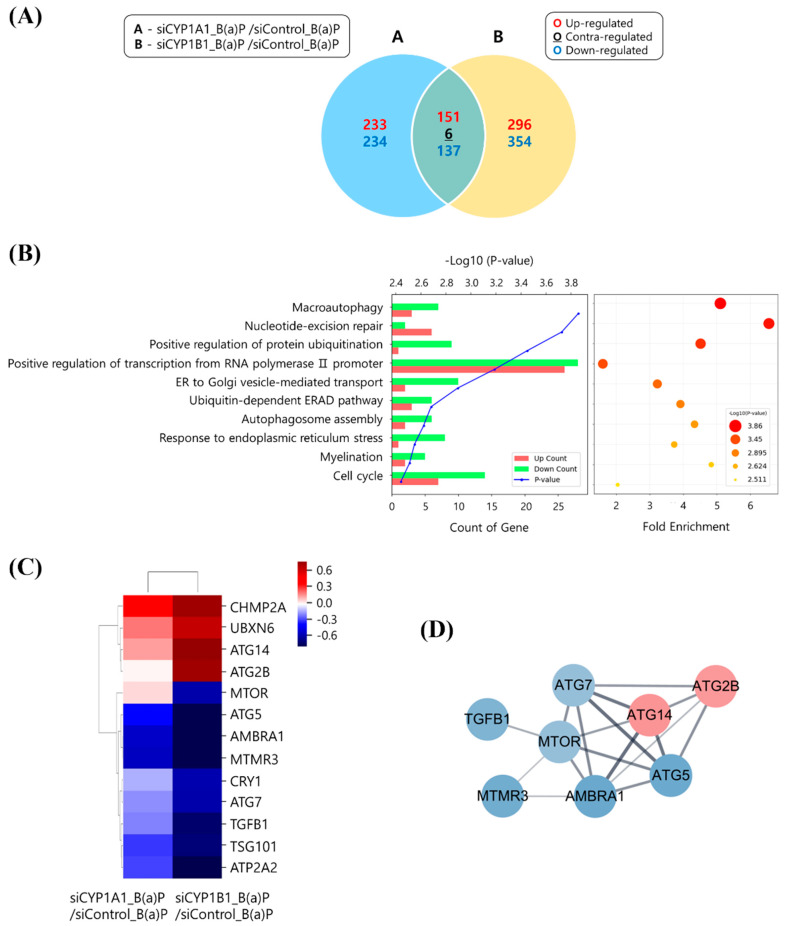
Gene expression related to macroautophagy is modulated by CYP1B1 knockdown. (**A**) The numbers of up- and down-regulated genes following CYP enzymes knockdowns are represented in the Venn diagram. The numbers of up- and down-regulated genes are shown in red and blue, respectively. The contra-regulated genes are shown in black. (**B**) Gene ontology (GO) and fold enrichment analyses were performed for about 650 genes, specifically regulated by CYP1B1 knockdown (fold change ≥ 1.5; normalized data (log2) ≥ 4). The top 10 categories are listed on the y-axis, with gene count and fold enrichment listed on the x-axis. (**C**) Hierarchical clustering heatmap was generated for 13 genes related to macroautophagy among CYP1B1-specific expression genes. Red and blue denote up- and down-regulation, respectively, and genetic distances indicate similar gene expression patterns. (**D**) Protein−protein interaction (PPI) analysis was performed. Up- or down-regulated genes are indicated by red or blue nodes, respectively. Lines connecting nodes represent gene interaction, with bold lines highlighting strong co-expression relationships. All experiments were performed in triplicate and repeated three times.

To determine how CYP1B1 knockdown reduces B[a]P-induced hepatic lipid accumulation and the genes that are involved, we knocked down the CYP enzymes and performed transcriptome analysis. After the knockdown of CYP1A1 and CYP1B1, the alterations in gene expression were analyzed and compared. Specifically, CYP1A1 knockdown led to changes in 467 genes, while CYP1B1 knockdown resulted in changes in 650 genes. Among the 650 genes that were specifically influenced by CYP1B1 knockdown, 296 and 354 genes were up- and downregulated, respectively (Figure 2A). We performed gene ontology (GO) analysis and gene fold enrichment analysis on the genes that were specifically modulated by CYP1B1 knockdown. We confirmed that the DEGs are related to ‘autophagosome assembly’, ‘response to endoplasmic reticulum stress’, and ‘cell cycle’, which are processes associated with macroautophagy (Figure 2B). This indicates that CYP1B1 plays a regulatory role in macroautophagy and lipid accumulation in hepatocytes. Subsequently, 13 macroautophagy-related DEGs were selected and utilized for hierarchical clustering heatmap and protein–protein interaction (PPI) analyses. Our analyses confirmed a significant reduction in the *mTOR* gene and identified genetic interactions between *mTOR* and macroautophagy-related genes after CYP1B1 knockdown (Figure 2C,D). These results suggest that CYP1B1 has a regulatory effect on the expression of macroautophagy-related genes, particularly *mTOR*.

**Figure 3:**
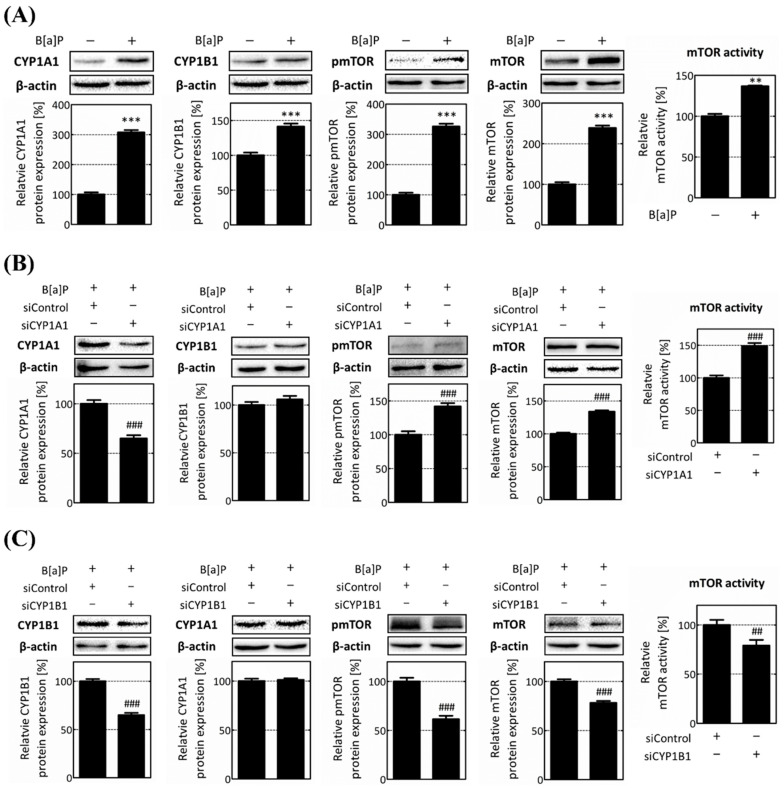
Protein level and the activity of mTOR are specifically reduced by CYP1B1. To verify the results obtained from transcriptome analysis following CYP enzymes knockdowns, mTOR expression and activity were analyzed at protein levels using Western blot analysis. (**A**) Increased protein levels of CYP1A1, CYP1B1, mTOR, and pmTOR after B[a]P treatment were investigated. (**B**) Protein levels of CYP1A1, CYP1B1, mTOR, and pmTOR after CYP1A1 knockdown were investigated. (**C**) Decreased protein levels of CYP1B1, CYP1A1, mTOR, and pmTOR following CYP1B1 knockdown were investigated. ** *p* < 0.01, *** *p* < 0.001, compared with the non-treated group; ## *p* < 0.01, ### *p* < 0.001, compared with the siControl group. B[a]P: benzo[a]pyrene (10 μM). The ratio between pmTOR and mTOR expression was calculated and expressed as mTOR activity. All experiments were performed in triplicate and repeated three times.

We further validated the results of the transcriptome analysis by analyzing the mRNA and protein expressions. The mRNA and protein levels of CYP1A1, CYP1B1, and mTOR were all increased following B[a]P treatment. Furthermore, the phosphorylated mammalian target of rapamycin (pmTOR) protein level was increased in the mTOR (Supplementary Figure S1A, Figure 3A). Following the knockdowns of CYP1A1 and CYP1B1, we verified the mTOR mRNA and protein levels. Specifically, in the CYP1A1 knockdown cell, the protein expressions of the mTOR and pmTOR were increased by 133.65% and 141.84%, respectively, and mTOR activity levels increased by 148.71% (Supplementary Figure S1B, Figure 3B). On the other hands, in the CYP1B1 knockdown cells, the mTOR mRNA and protein levels decreased 0.34-fold and to 78.04%, respectively. Additionally, the pmTOR protein level and mTOR activity were also reduced by 61.53% and 78.94%, respectively (Supplementary Figure S1C, Figure 3C). Overall, these data are consistent with the transcriptome analysis, and we confirmed that mTOR is specifically reduced by CYP1B1 knockdown, independently of CYP1A1. Our results suggest that CYP1B1 influences the expression of macroautophagy-related genes, which particularly impact mTOR, regardless of CYP1A1.

### 2.3. CYP1B1 Knockdown Mitigates Lipid Accumulation by Enhancing Intracellular Lipophagy, Which Is Reduced by B[a]P

**Figure 4:**
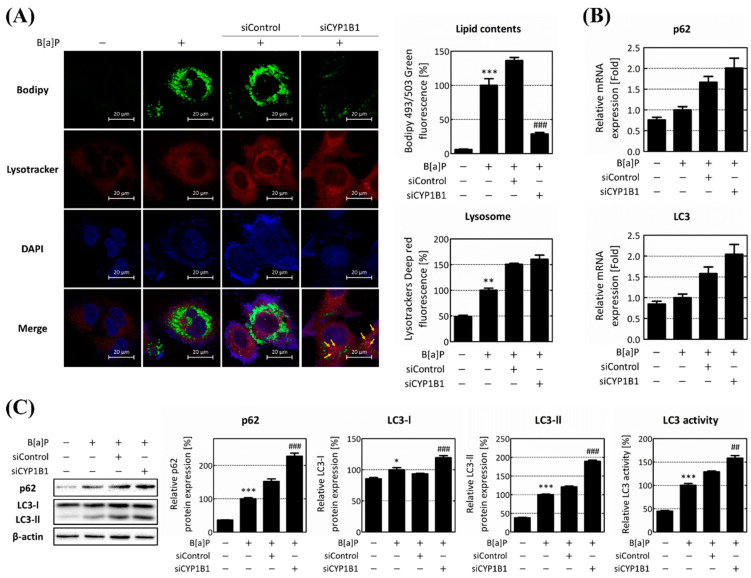
CYP1B1 knockdown induces lipophagy by enhancing phagolysosome biogenesis. (**A**) Intracellular lipid accumulation, lysosome, and phagolysosome generation were assessed using Bodipy 493/503 and Lysotracker fluorescence measurements. Lipid contents were calculated by measuring green fluorescence, representing intracellular LDs, while lysosome was quantified using red fluorescence. And the co-localization of lipids and lysosomes was indicated by yellow arrows. (**B**) The mRNA and (**C**) protein expression levels of autophagic flux genes, p62 and LC3, were analyzed by RT-qPCR and Western blot analysis following B[a]P treatment and CYP1B1 knockdown. * *p* < 0.05, ** *p* < 0.01, *** *p* < 0.001, compared with the non-treated group; ## *p* < 0.01, ### *p* < 0.001, compared with the siControl group. B[a]P: benzo[a]pyrene (10 μM). The expression ratio of LC3-II and LC3-I was calculated and expressed as LC3 activity. All experiments were performed in triplicate and repeated three times.

To explore the correlation between CYP1B1 and B[a]P-induced hepatic lipid accumulation, we focused on lipophagy involved in lipid metabolism and macroautophagy. To validate the intracellular lipophagy, CYP1B1 knockdown was performed, and the formation of phagolysosomes and its relevant gene expressions were analyzed. Intracellular lipids increased dramatically within the B[a]P-treated group. However, following CYP1B1 knockdown, they decreased to 21.09% compared to the siControl group. In the B[a]P-treated group, lysosome increased to 203.74% compared to the non-treated group. In the siControl group and CYP1B1 knockdown group, it increased to 150.61% and 160.43% compared to the B[a]P-treated group. The co-localization of the two fluorescent signals indicated that phagolysosomes were generated during lipophagy, which is indicated by the yellow arrows. Co-localization was increased in CYP1B1 knockdown group compared to the B[a]P-treated group and siControl group (Figure 4A). These data indicate that phagolysosome formation and the progression of lipophagy occur following CYP1B1 knockdown.

Furthermore, the overall progress of lipophagy protein expression levels of autophagic flux genes, p62 and LC3, was investigated. The mRNA levels of *p62* and *LC3* increased 1.32-fold and 1.18-fold, respectively, after B[a]P treatment. Following CYP1B1 knockdown, the mRNA levels of *p62* and *LC3* both increased over 1.2-fold compared to the siControl group (Figure 4B). Moreover, the protein level of p62 increased to 152.73% compared to the siControl group following CYP1B1 knockdown. Additionally, CYP1B1 knockdown increased the protein levels of both LC3-I and LC3-II to 131.53% and 155.35%, respectively, compared to the siControl group. The ratio of LC3-II to LC3-I, which acts as an indicator of LC3 activity and autophagosome production, also increased to 118.11%, in comparison to the siControl group (Figure 4C). The intracellular lipid content decreased upon CYP1B1 knockdown, whereas the expression of the autophagic flux genes increased. These results suggest that CYP1B1 plays a pivotal role in regulating lipophagy by influencing phagolysosome formation and the expressions of the genes involved in the autophagic flux.

### 2.4. CYP1B1 Knockdown Enhances Lipophagy via the Induction of TFE3 Translocation

**Figure 5:**
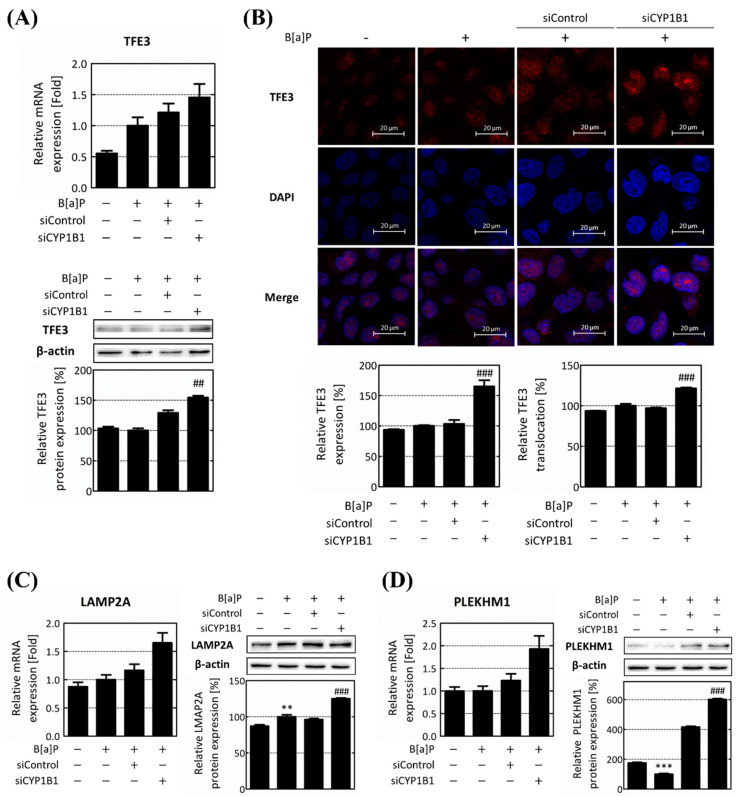
CYP1B1 knockdown induces TFE3 nuclear translocation and its target gene expression. (**A**) mRNA and protein expression levels of TFE3 were detected using RT-qPCR and Western blot analysis, respectively. (**B**) TFE3 expression and nuclear translocation levels were visualized using immunofluorescence analysis. The TFE3 expression level was quantified based on the red fluorescence of the entire cell. The translocation of TFE3 was calculated as the ratio of red fluorescence values in the whole cells and nucleus. mRNA and protein expression levels of TFE3 downstream target genes, (**C**) LAMP2A and (**D**) PLEKHM1, were confirmed using RT-qPCR and Western blot analysis following B[a]P treatment and CYP1B1 knockdown. ** *p* < 0.01, *** *p* < 0.001, compared with the non-treated group; ## *p* < 0.01, ### *p* < 0.001, compared with the siControl group. B[a]P: benzo[a]pyrene (10 μM). The ratio between pmTOR and mTOR expression was calculated and expressed as mTOR activity. All experiments were performed in triplicate and repeated three times.

To confirm the regulatory role of B[a]P-induced CYP1B1 expression on lipophagy, the expression levels of TFE3 and its downstream target genes were evaluated. Upon CYP1B1 knockdown, both the TFE3 mRNA and protein levels exhibited increments, reaching 1.2-fold and 117.05%, respectively, in comparison to the siControl group (Figure 5A). We further confirmed the intracellular expression of TFE3 and its nuclear translocation by immunofluorescence analysis. Upon CYP1B1 knockdown, the TFE3 expression increased to 165.09% compared to the B[a]P-treated group and 159.89% compared to the siControl group. Thereafter, the TFE3 nuclear translocation was quantified as the proportion within the nucleus relative to the total intracellular TFE3 content. The TFE3 translocation was confirmed as being 93.68%, 100%, 97%, and 121.45% in the non-treated group, B[a]P-treated group, siControl group, and CYP1B1 knockdown group, respectively. Following CYP1B1 knockdown, the TFE3 nuclear translocation escalated to 129.64% and 125.20%, compared to the non-treated group and siControl group, respectively (Figure 5B). Subsequently, we validated the expression levels of the lipophagy-related genes, which were located downstream of TFE3. The mRNA and protein levels of the lysosomal-associated membrane protein 2A (LAMP2A) also increased following CYP1B1 knockdown, exhibiting 1.42-fold and 131% increases compared to the siControl group (Figure 5C). Similarly, the expression level of pleckstrin homology and RUN domain containing M1 (PLEKHM1) significantly increased upon CYP1B1 knockdown (Figure 5D). These results emphasize that CYP1B1 knockdown augments TFE3 expression and translocation, consequently elevating the expression of downstream lipophagy-related genes, and ultimately resulting in the up-regulation of lipophagy.

### 2.5. CYP1B1-Mediated Regulation of mTOR Amplifies Hepatic Lipid Accumulation via Lipophagy Suppression

**Figure 6:**
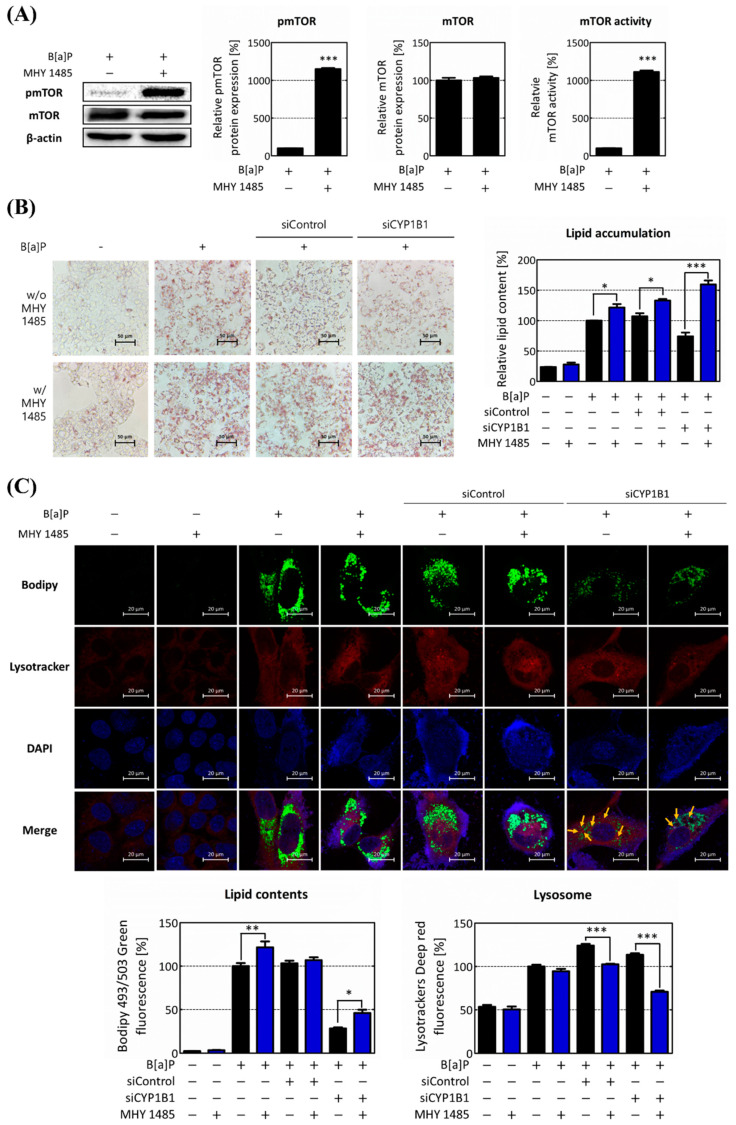
Up-regulation of mTOR activity increases intracellular lipid accumulation and reduces lipophagy. To investigate the role of mTOR in regulating lipophagy, its effects were assessed through treatment with the mTOR activator, MHY 1485. (**A**) mTOR expression level and activity were analyzed following MHY 1485 treatment. (**B**) The increase in lipid accumulation after MHY 1485 treatment was confirmed using ORO staining. (**C**) Intracellular lipid accumulation, lysosome, and phagolysosome production were assessed using Bodipy and Lysotracker after MHY 1485 treatment. Intracellular neutral lipid is represented in the graph by calculating the intracellular green fluorescence, and lysosome is expressed in the graph by red fluorescence. Yellow arrows indicate the co-localization of lipids and lysosomes * *p* < 0.05, ** *p* < 0.01, *** *p* < 0.001, compared with each MHY 1485 non-treated group. B[a]P: benzo[a]pyrene (10 μM); MHY 1485: mTOR activator (2 μM). w/o MHY 1485: treat without MHY 1485; w/MHY 1485: treat with MHY 1485. The blue bars indicate the treatment of MHY 1485. All experiments were performed in triplicate and repeated three times.

The previous results clearly established that CYP1B1 influences lipid accumulation within hepatocytes through the regulation of intracellular lipophagy. Significantly, mTOR, an entity under the control of CYP1B1, plays a central role in orchestrating these regulatory phenomena. Therefore, to confirm these findings, we administered the mTOR activator MHY 1485 and performed a series of analyses to verify our previous observations. The expression of mTOR exhibited no significant changes upon MHY 1485 treatment. However, the pmTOR expression and mTOR activity increased dramatically upon MHY 1485 treatment to 1149.27% and 1111.43%, respectively (Figure 6A). Therefore, these results indicate that MHY 1485 increases mTOR activity by regulating the phosphorylation of mTOR rather than its expression.

Next, we investigated intracellular lipid content following the increase in mTOR activity. It was confirmed that lipid content increased following MHY 1485 treatment (Figure 6B). Subsequently, lipophagy was measured through phagolysosome generation in the response to the MHY 1485 treatment. The neutral lipid accumulation was enhanced in all groups by the increase in the mTOR activity. Remarkably, in the CYP1B1 knockdown group, the co-localization of lipids and lysosomes was reduced upon MHY 1485 treatment (Figure 6C). Therefore, these results suggest that the progression of lipophagy is inhibited by an increase in mTOR activity. This strongly implied that mTOR and its regulation by CYP1B1 play a pivotal role in governing intracellular lipid accumulation, particularly relating to lipophagy among lipid regulation mechanisms.

**Figure 7:**
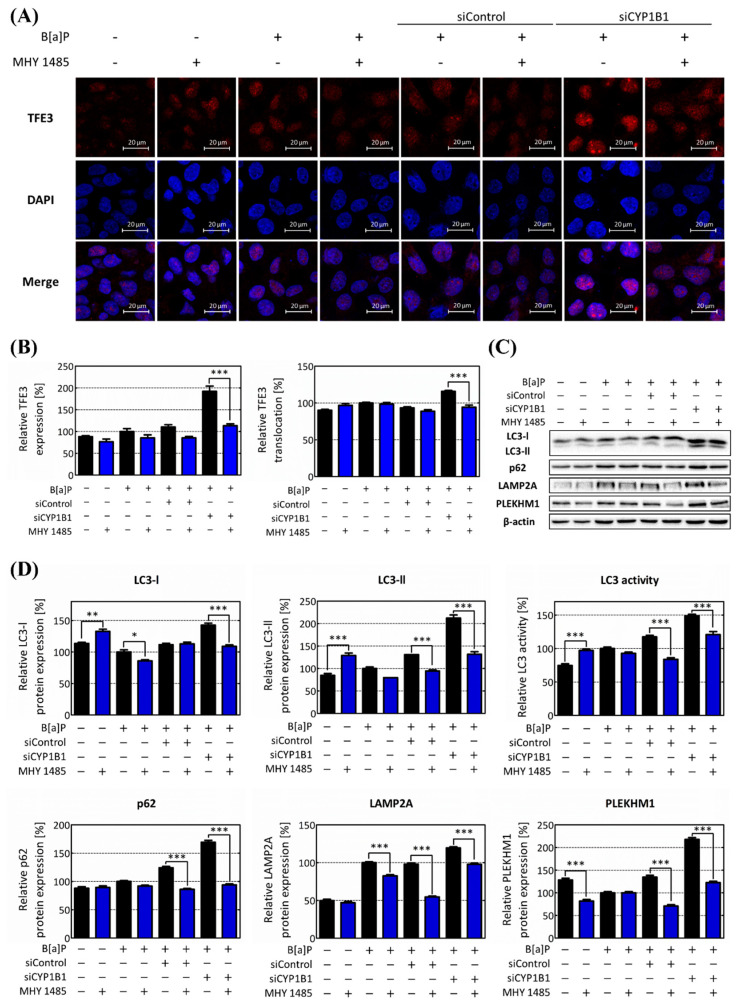
mTOR decreases lipophagy through the attenuation of TFE3 nuclear translocation. (**A**) The TFE3 expression levels and their nuclear translocation in response to the up-regulation of mTOR activity were assessed through immunofluorescence analysis. (**B**) The red fluorescence values were quantified, and the overall cell TFE3 expression was graphed. The ratio of red fluorescence in whole cells to nuclei represents the translocation of TFE3, which is shown in the graph. (**C**) Protein levels of autophagic flux genes (LC3 and p62) and TFE3 downstream genes (PLEKHM1 and LAMP2A) following the up-regulation of mTOR activity were confirmed using Western blot analysis. (**D**) The relative expression levels of genes associated with lipophagy progression were calculated and graphed. * *p* < 0.05, ** *p* < 0.01, *** *p* < 0.001, compared with each MHY 1485 non-treated group. The expression ratio of LC3-II and LC3-I was calculated and expressed as LC3 activity. B[a]P: benzo[a]pyrene (10 μM); MHY 1485: mTOR activator (2 μM). The blue bars indicate the treatment of MHY 1485. All experiments were performed in triplicate and repeated three times.

Subsequently, TFE3 expression and its translocation levels as well as the gene expression of its downstream targets were investigated following MHY 1485 treatment. Here, MHY 1485 treatment significantly reduced the expression of TFE3. TFE3 translocation was also reduced by MHY 1485 treatment. (Figure 7A,B). Furthermore, MHY 1485 treatment also reduced the protein levels of the autophagic flux genes and TFE3 downstream target genes. In the case of LC3, both LC3-I and LC-II were decreased by MHY 1485 treatment. The expression of p62 decreased to 55.48% in the CYP1B1 knockdown group, compared to the MHY 1485 untreated group. In the case of LAMP2A, MHY 1485 treatment reduced its expression. MHY 1485 treatment also decreased the expression of PLEKHM1 in the CYP1B1 knockdown group to 56.26% (Figure 7C,D). As mTOR activity increased, the expression and translocation of TFE3 decreased, and the expressions of lipophagy-related genes were also suppressed. These results confirmed that mTOR is a negative regulator of lipophagy and acts as a major factor in B[a]P-induced lipid accumulation in hepatocytes.

## 3. Discussion

The global prevalence of MASLD is currently estimated at 32.4% and is expected to continue to increase [2,39]. However, despite the emerging severity of MASLD, the mechanisms underlying its development are still poorly understood. Therefore, studies are needed to elucidate the pathogenesis related to MASLD. Indeed, several factors are known to contribute to the development of MASLD, including PAH exposure: the prominent example is B[a]P [40]. People are easily exposed to B[a]P in daily life through cigarette smoke and food intake. According to the World Health Organization and IARC, the amount of B[a]P contained in cigarette smoke has been reported to be 52–95 ng/cigarette, and it is known that each person consumes several nanograms to micrograms through food per day [41]. B[a]P induces the expression of CYP enzymes, leading to lipid accumulation in hepatocytes. However, the precise mechanism for this process remains unclear [16,19]. Therefore, in this study, we aimed to investigate the relationship between B[a]P-induced hepatic lipid accumulation and CYP1B1, to determine the pivotal mechanisms and factors for its action.

Environmental exposure to B[a]P is known to induce lipid accumulation in hepatocytes. Therefore, we first validated lipid accumulation in hepatocytes following B[a]P treatment. Our findings confirmed that hepatic lipid contents increased after B[a]P treatment (Figure 1A). Through Figure 1A, the optimal B[a]P concentration for the study was selected as 10 μM that maximizes lipid accumulation in HepG2 cells. Additionally, we confirmed the lipid contents following CYP1B1 knockdown to analyze the effect of CYP1B1 on hepatic lipid accumulation. The results show that B[a]P-induced lipid accumulation was reduced upon CYP1B1 knockdown (Figure 1B).

Generally, primary human hepatocytes (PHHs) induce pluripotent stem cell (iPSC)-derived hepatocytes, and hepatic carcinoma cell lines can be used in in vitro studies involved in hepatic lipid accumulation. They are known to be the most similar to the phenotype of hepatocytes [42]; however, their phenotype is unstable and their culture environment is limited [43]. In contrast, HepG2 cells maintain a stable phenotype throughout the culture period, exhibit similarities to the human liver, and are known to significantly induce CYP enzymes in response to xenobiotics [21,44,45,46]. Therefore, our study was performed using the HepG2 cell model, considering cell stability and CYP enzymatic expressions. The results in Figure 1 confirm that B[a]P induces lipid accumulation in HepG2 cells and that CYP1B1 plays a major role in this process.

To determine the pathways and genes that are affected by CYP1B1, a transcriptome analysis was conducted. Among the genes specifically changed by CYP1B1 knockdown, the macroautophagy-related genes were the most affected (Figure 2A,B). Macroautophagy has some subcategories, and the pathway related to lipid degradation is called lipophagy. Recent studies have shown and emphasized that a relationship between lipophagy and hepatic lipid accumulation does exist. Indeed, higher levels of negative regulators of lipophagy were observed in the livers of MASLD patients with steatosis compared to those without steatosis, and impaired lipophagy has been linked to endoplasmic reticulum (ER) stress and MASLD development [47]. Consequently, lipophagy could be a new target for the development of MASLD [30,48]. These results suggest that the reduction in hepatic lipid contents induced by CYP1B1 knockdown is affected by lipophagy, a type of macroautophagy.

To elucidate the genes that play an important role in this pathway, the DEGs involved in macroautophagy were selected and analyzed. Notably, mTOR expression was increased by CYP1A1 knockdown, yet it was only significantly reduced following CYP1B1 knockdown (Figure 2C,D). The mTOR mRNA and protein expressions, which were increased by B[a]P treatment, were further increased upon CYP1A1 knockdown, although, contrastingly, they were significantly decreased by CYP1B1 knockdown (Supplementary Figure S1, Figure 3). These results demonstrate that mTOR is specifically regulated by CYP1B1. mTOR is also well known to inhibit lipophagy by affecting genes involved in the induction of autophagosomes [34,35,49]. In nutrient-rich conditions, activated mTOR suppresses the formation of autophagosomes. Conversely, when nutrients are insufficient, mTOR is inactivated, which induces autophagosome formation [33,35]. Although the importance of mTOR in lipid metabolism is gradually emerging, research on the exact mechanisms of its involvement is still insufficient. Therefore, this study focused on mTOR-related mechanisms affecting the B[a]P-induced hepatic lipid accumulation and lipophagy. Then, the ratio between pmTOR and mTOR expression was calculated and expressed as mTOR activity. Over the various B[a]P treatment durations, the pmTOR and mTOR expressions peaked at 24 h (Supplementary Figure S3). The results illustrated in Figure 2 and Figure 3 demonstrate that mTOR was increased upon CYP1A1 knockdown but dramatically decreased upon CYP1B1 knockdown. These results show that CYP1B1-mediated changes in mTOR are a more major response, and thus mTOR is specifically reduced by CYP1B1 knockdown.

First, to validate the impact of CYP1B1 on the lipophagy process, we scrutinized alterations in the lysosome and phagolysosome production. The intracellular lipids decreased upon CYP1B1 knockdown. And the lysosome increased after B[a]P treatment and further increased following CYP1B1 knockdown. However, the co-localization of lipids and lysosomes, which indicates the production of phagolysosomes, was more noticeable upon CYP1B1 knockdown (Figure 4A). Our results strongly suggest that CYP1B1 knockdown promotes the production of phagolysosomes, ultimately leading to a reduction in the lipid levels within hepatocytes. p62 and LC3 are representative autophagic flux genes. p62 induces autophagy through the movement, assembly, and sequestration of LDs [50]. LC3 is converted from LC3-I to LC3-II and recruited to the autophagosome membrane, while autophagosomes engulf the organelles in the cytoplasm [51]. The expression levels of both p62 and LC3 increased dramatically upon CYP1B1 knockdown (Figure 4B,C). The results in Figure 4 demonstrate that CYP1B1 reduces lipophagy in hepatocytes and increases overall lipid contents.

Subsequently, to elucidate the regulatory role of CYP1B1 expression in the lipophagy process, the expression of TFE3 and its downstream target genes were investigated. The MiTF/TFE family, including TFE3, which is a key regulator in autophagy, is regulated by mTOR. Typically, the MiTF/TFE family remains suppressed by mTOR and is confined to the cytoplasm [34]. However, upon mTOR inactivation, these proteins translocate to the nucleus and initiate the expression of lipophagy-related genes [52]. Therefore, in this study, we analyzed whether CYP1B1 knockdown affected the entire lipophagy process by up-regulating the expression and translocation of TFE3. After CYP1B1 knockdown, the expression of TFE3 was up-regulated, while TFE3 translocation was significantly increased (Figure 5A,B). Furthermore, the expressions of lipophagy-related genes, which exist downstream of TFE3, were confirmed following CYP1B1 knockdown. LAMP2A promotes lysosome activity, and PLEKHM1 is involved in the generation and maturation of phagolysosomes [53,54]. LAMP2A is well known as a chaperone-mediated autophagy (CMA)-related gene, but this gene is regulated by the MiTF/TFE family and is known to play an important role in lipid homeostasis [55,56]. The expressions of LAMP2A and PLEKHM1 were increased upon CYP1B1 knockdown (Figure 5C,D). Therefore, it was confirmed that CYP1B1 induced by B[a]P participates in hepatic lipid accumulation by regulating lipophagy.

Finally, to validate whether this pathway is related to mTOR, which is specifically regulated by CYP1B1, we performed additional analyses using the mTOR activator. MHY 1485 is a well-established mTOR activator known to enhance mTOR activity by regulating the expression level of pmTOR [57,58]. Upon MHY 1485 treatment, the pmTOR expression and mTOR activity both increased rapidly (Figure 6A). Moreover, following MHY 1485 treatment, the intracellular lipid contents increased, whereas the lysosome and phagolysosome production decreased (Figure 6B,C). In addition, the expression and translocation of TFE3 were both suppressed, and the expressions of lipophagy-related genes were also reduced by MHY 1485 treatment (Figure 7). These results validate that mTOR suppresses lipophagy by regulating TFE3 and its related downstream genes following B[a]P-induced hepatic lipid accumulation. The inhibitory effect of MHY 1485 on phagolysosome generation has been confirmed in previous studies [59]. In addition, according to Seok et al., 2021, MHY 1485 induces the phosphorylation of mTOR, resulting in a decrease in intracellular LC3, especially the LC3-II form [60]. Therefore, it has been proven that MHY 1485 inhibits autophagy by activating mTOR. Furthermore, our study demonstrated that MHY 1485 further increases B[a]P-induced lipid accumulation in hepatocytes by inhibiting lipophagy. Moreover, it can be said that mTOR regulates hepatic lipid accumulation when only CYP1B1 is induced by B[a]P. Therefore, the results in Figure 6 and Figure 7 show that mTOR is an important regulator of the lipophagy pathway in B[a]P-induced hepatic lipid accumulation.

There are certain limitations in our study. Firstly, our investigation predominantly focused on the mechanistic aspects of B[a]P-induced hepatic lipid accumulation and its association with the mTOR-regulated lipophagy pathway. While the identified mechanism provides valuable insights, the broader implications and potential therapeutic strategies warrant further exploration. Secondly, the experimental design primarily employed cell culture models and molecular analyses. While these systems offer controlled environments for mechanistic investigations, translating these findings to in vivo scenarios and clinical applications necessitates caution. Animal models and, eventually, human studies are imperative to validate our observations’ relevance and potential translatability.

In conclusion, our research revealed a novel mechanism that B[a]P-induced hepatic lipid accumulation is mediated by the suppression of the mTOR-regulated lipophagy pathway, a major target of CYP1B1. These results emphasize the potential of inducing mTOR using CYP1B1 as a novel therapeutic target for comprehending and managing B[a]P-induced MASLD. Additional research is needed to comprehensively understand the complexity of this phenomenon and develop targeted therapeutic interventions.

## 4. Materials and Methods

### 4.1. Reagents

B[a]P, dimethyl sulfoxide (DMSO), MHY 1485, ORO, formaldehyde solution, isopropanol, and 4,6-diamidino-2-phenylindole dihydrochloride (DAPI) were purchased from Sigma-Aldrich (St. Louis, MO, USA). Bodipy 493/503 and Lysotracker Deep Red were purchased from Thermo Fisher Scientific (Waltham, MA, USA). CYP1B1 polyclonal antibody (18505-1-AP), phospho-mTOR (Ser2448) mouse monoclonal antibody (67778-1-IG), TFE3 polyclonal antibody (14480-1-AP), and LC3 polyclonal antibody (14600-1-AP) were purchased from Proteintech (Rosemont, IL, USA). mTOR (7C10) rabbit mAb (2983s), PLEKHM1 antibody (66012s), and anti-rabbit IgG, HRP-linked antibody (7074s) were purchased from Cell Signaling Technology (Danvers, MA, USA). β-actin (C-4) (sc-47778), CYP1A1 (A-9) (sc-393979), and m-IgGκ BP-HRP (sc-516102) were purchased from Santa Cruz Biotechnology (Dallas, CA, USA). LAMP-2A polyclonal antibody (AMC2) (51-2200) and SQSTM1 (P62) polyclonal antibody (PA5-20839) were purchased from Thermo Fisher Scientific (Waltham, MA, USA). Goat anti-rabbit IgG antibody (DyLight594) (GTX213110-05) was purchased from GeneTex (Irvine, CA, USA).

### 4.2. Cell Culture and Treatment

The human hepatic carcinoma cell line HepG2 was purchased from the American Type Culture Collection (ATCC; Manassas, VA, USA). Cells with a passage number between 11 and 15 were used and cultured in a 150 mm^2^ cell culture dish. Cells were cultured in Roswell Park Memorial Institute (RPMI) 1640 medium (Welgene, Gyeongsan, Republic of Korea) supplemented with 10% fetal bovine serum (FBS; Gibco, Grand Island, NY, USA), 100 μg/mL penicillin/streptomycin (Welgene, Gyeongsan, Republic of Korea), and 1 mM sodium pyruvate (Welgene, Gyeongsan, Republic of Korea) in a humidified incubator at 37 °C with 5% CO_2_. The culture medium was changed every 3 days. For the assays, cells were treated with or without B[a]P and transfected with CYP1A1, CYP1B1, or control siRNA for 24 h or 48 h. A 10 mM stock solution of B[a]P in DMSO was diluted to a final concentration of 10 μM in cell culture media. Moreover, in the case of MHY 1485 (Sigma-Aldrich Chemical, St. Louis, MO, USA), an mTOR activator, it was additionally treated at a concentration of 2 μM during the transfection process and cultured for 24 h or 48 h.

### 4.3. siRNA Transfection

HepG2 cells were plated in 6-well plates at a density of 60 × 10^4^ cells/well, using antibiotic-free RPMI 1640 medium supplemented with 10% FBS. Concurrently, the cells were transfected with 50 pM of CYP1A1, CYP1B1, or control siRNA (Genolution, Seoul, Republic of Korea) using Lipofectamine 3000 (Invitrogen, Waltham, MA, USA), following the manufacturer’s protocol. Briefly, the transfection process involved mixing 50 pM siRNA with 100 μL of Lipofectamine 3000 dissolved in the culture medium. Subsequently, the cells were incubated for 24 h or 48 h before being utilized for assays. For CYP1A1 and CYP1B1 siRNAs, five types of siRNAs were compared and the siRNAs for CYP1A1-1 and CYP1B1-2, which showed the highest knockdown efficiencies, were selected (Supplementary Figure S4). The sequences for all siRNAs are listed in Supplementary Table S1, with the sequences for the control, CYP1A1, and CYP1B1 siRNAs listed again in Table 1.

### 4.4. Cell Viability Assay

Cell viability assay was performed to assess the toxicity of B[a]P, siRNA, and MHY 1485 to cells. HepG2 cells were seeded in 24-well plates at a density of 10 × 10^4^ cells/well. Following transfection and a 48 h treatment period, the culture medium was removed, and the cells were washed with PBS. Next, RPMI 1640 medium containing Quanti-Max WST-8 Cell Viability Assay Solution (WST-8 Solution, Biomax, Seoul, Republic of Korea) was added and the cells were incubated at 37 °C for 30 min. After incubation, 95 μL of the supernatant was transferred in triplicate from each well to a 96-well plate. The absorbance was measured at 450 nm using a Sunrise™ Absorbance microplate reader (TECAN, Männedorf, Switzerland).

### 4.5. Lipid Accumulation Test Using ORO Staining

ORO staining was conducted to confirm the accumulation of LDs in HepG2 cells. The cells were seeded and transfected in 6-well plates at a density of 60 × 10^4^ cells/well and allowed to incubate for 48 h. Following an incubation period, the cells were washed twice with PBS and fixed using 4% formaldehyde (Sigma-Aldrich, St. Louis, MO, USA) for 15 min. Subsequently, the cells were washed with distilled water (DW) and left to air-dry at room temperature. Cells were stained with 0.3% ORO solution (Sigma-Aldrich, St. Louis, MO, USA) for 20 min at room temperature in the dark. Then, the cells were washed with 60% isopropanol (Sigma-Aldrich, St. Louis, MO, USA). To quantify the intracellular LDs staining by the ORO solution, the dye was dissolved in 100% isopropanol and placed on a shaker at 70 rpm for 20 min in the dark. Following the incubation, 100 μL of supernatant was transferred to each well in a 96-well plate, and the absorbance was measured at 492 nm by a Sunrise™ Absorbance microplate reader (TECAN, Männedorf, Switzerland). Each result was corrected using the cell viability value (Supplementary Figure S2).

### 4.6. mRNA Extraction

Total mRNA was extracted for mRNA quantification sequencing (Quan-Seq) and cDNA synthesis. HepG2 cells were seeded and transfected in a 6-well plate. Subsequently, the cells were washed twice with PBS, and total RNA was extracted using TRIzol reagent (Invitrogen, Waltham, MA, USA), in accordance with the manufacturer’s protocol. Then, the isolated RNA was dissolved in RNase-free water. For quality assessment, the RNA concentration and purity were measured using Nanodrop 2000 (Thermo Fisher Scientific, Waltham, MA, USA), and RNA samples with absorbance of >1.8 at 260 and 280 nm were deemed suitable for further use.

### 4.7. mRNA Quantification Sequencing (Quan-Seq)

mRNA Quan-Seq analysis was performed by E-Biogen Inc. (Seoul, Republic of Korea). Each RNA sample library was built using the QuantSeq 3′ mRNA-Seq Library Prep Kit FWD for Illumina (Lexogen, Vienna, Austria). For data visualization, the Excel-based Differentially Expressed Gene Analysis (ExDEGA; E-Biogen, Inc., Seoul, Republic of Korea) was used. To gain insight into the functional characteristics of the differentially expressed genes (DEGs, fold change ≥ 1.5; normalized data (log_2_) ≥ 4), a GO analysis was performed. The genes were categorized and grouped using DAVID (https://david.ncifcrf.gov/, accessed on 23 February 2023). In addition, hierarchical clustering and PPI analysis were performed to validate the expression patterns and functional interaction network of the genes. For predicting protein interaction, the Cytoscape STRING database (http://string-db.org/, accessed on 23 February 2023) was utilized. Finally, to present the findings effectively, each graph was visualized using ExDEGA Graphic Plus 2.0 (E-Biogen, Inc., Seoul, Republic of Korea).

### 4.8. Reverse Transcription-Quantitative Polymerase Chain Reaction (RT-qPCR)

The expression of macroautophagy-related genes was analyzed using RT-qPCR. cDNA was synthesized from 2000 ng of RNA through reverse transcription, using M-MLV reverse transcriptase (ELPIS-BIOTECH, Daejeon, Republic of Korea). For the RT-qPCR, the SYBR Green PCR master mix (KAPA Biosystems, Wilmington, MA, USA) and real-time PCR detection system (BIO-RAD, Hercules, CA, USA) were used. The reactant mixture comprised 1 μL of template cDNA, 1 μL each of forward and reverse primer (5 μM each), 7 μL of DW, and 10 μL of SYBR Green PCR master mix. The reaction conditions involved an initial pre-denaturation step at 95 °C for 5 min, followed by 50 cycles of denaturation at 95 °C for 10 s, annealing at 60 °C for 30 s, and extension at 72 °C for 30 s. To ensure a relative comparison, all genes were normalized against the β-actin gene, and the primer sequences of the genes used in this analysis are listed in Table 2.

### 4.9. Western Blot Analysis

Protein expression levels were analyzed through Western blot analysis. Cells were lysed using RIPA lysis buffer (Bio Solution, Seoul, Republic of Korea) containing a protease inhibitor cocktail (Sigma-Aldrich Chemical, St. Louis, MO, USA), and a phosphatase inhibitor cocktail 2/3 (Sigma-Aldrich Chemical, St. Louis, MO, USA). After the cells were lysed, total protein was collected from the cell lysates through centrifugation at 20,000× *g* and 4 °C for 15 min. Protein quantification was performed using the BCA protein assay kit (Thermo Fisher, Waltham, MA, USA), in accordance with the manufacturer’s instructions. Equal amounts of protein were loaded onto a 10% sodium dodecyl sulfate-polyacrylamide gel electrophoresis (SDS-PAGE) gel for each sample. Subsequently, the separated proteins were transferred to polyvinylidene difluoride (PVDF) membranes (Millipore, Burlington, MA, USA). To prevent non-specific binding, the membranes were blocked with 5% skim milk at room temperature for 1 h. Then, the membranes were incubated with the specified primary antibodies in 2.5% bovine serum albumin (BSA) solution containing sodium azide (Sigma-Aldrich Chemical, St. Louis, MO, USA), followed by secondary antibodies in 1% skim milk. Target proteins were detected using ECL Plus Western blotting detection reagent (Amersham Bioscience, Buckinghamshire, UK), using a Bio-Rad ChemiDoc XRS System (Hercules, CA, USA). The obtained data were quantified using Quantity One imaging software (Bio-Rad, Hercules, CA, USA), and comparisons were performed using β-actin as an internal control. Also, the expression ratio of LC3-II and LC3-I was calculated and expressed as LC3 activity, and the ratio between pmTOR and mTOR expression was calculated and expressed as mTOR activity.

### 4.10. Biogenesis of Phagolysosomes

The accumulation of intracellular LDs and the progression of lipophagy were verified by observing changes in phagolysosome production. Before cell seeding, a 22 × 22 mm coverslip was placed in each well of a 6-well plate and sterilized with 90% ethanol and UV light. Subsequently, the coverslip was coated with 0.01% poly-L-lysine (Sigma-Aldrich Chemical, St. Louis, MO, USA) for 30 min. Afterward, each coverslip was washed twice with DW and air-dried overnight. Then, HepG2 cells were seeded and transfected on the prepared coverslip and allowed to incubate for 48 h. Following the incubation, the culture medium was removed, and the cells were washed twice with PBS before being fixed with 4% formaldehyde for 15 min. The staining process followed in the sequence of Bodipy 493/503, Lysotracker Deep Red, and DAPI. To conduct the staining, 1 μg/mL Bodipy 493/503 was treated at 37 °C for 1 h. The cells were washed twice with ice-cold PBS and then a 100 nM Lysotracker Deep Red solution was added for 1 h at 37 °C. The cells were washed twice with ice-cold PBS and 1 μg/mL DAPI was used for 1 min to stain the cells at room temperature. The cells were mounted onto a glass slide using a mounting medium (Dako, Glostrup Kommune, Denmark). The fluorescence images were captured using confocal microscopy (Carl Zeiss, Oberkochen, Germany) and were quantified using ImageJ software ver. 1.8.0 (National. Institutes of Health, Bethesda, MD, USA). The ‘Bodipy 493/503 Green fluorescence [%]’ and ‘Lysotracker Deep Red fluorescence [%]’ were quantified using the following calculation (intensity of green or red fluorescence/intensity of DAPI fluorescence).

### 4.11. Immunofluorescence Analysis

The nuclear translocation of TFE3 was validated through immunofluorescence analysis. Before cell seeding, a coverslip sized 22 × 22 mm was placed in each well of a 6-well plate. Subsequently, sterilization and coating with poly-L-lysine were performed, as described above. Then, HepG2 cells were seeded and transfected on the coverslips, and following an incubation period of 48 h, the cells were washed twice with ice-cold PBS. The fixation step was performed using 4% formaldehyde for 15 min, followed by permeabilization using 0.25% TritonX-100 (Sigma-Aldrich, St. Louis, MO, USA) for 10 min. Then, blocking was performed using 1% bovine serum albumin (BSA; Sigma-Aldrich, St. Louis, MO, USA). The cells were sequentially incubated with primary and secondary antibodies, each for 1 h, at room temperature. The primary antibody used for the analysis was TFE3 polyclonal antibody (14480-1-AP), which was diluted 1:250 in 1% BSA. The secondary antibody, Goat anti-rabbit IgG antibody (DyLight594) (GTX213110-05), was diluted 1:1000 in 1% BSA. To visualize the nuclei, the cells were stained with DAPI (Sigma-Aldrich, St. Louis, MO, USA) at a concentration of 1 μg/mL in PBS for 1 min at room temperature. Finally, the cells were mounted using a mounting medium (Dako, Glostrup Kommune, Denmark). Fluorescence images were captured using confocal microscopy (Carl Zeiss, Oberkochen, Germany) and were quantified using ImageJ software ver. 1.8.0 (National Institutes of Health, Bethesda, MD, USA). The ‘Relative TFE3 translocation [%]’ was quantified using the calculation (fluorescence intensity of nuclei/fluorescence intensity of cell).

### 4.12. Statistical Analysis

All statistical analyses were performed using GraphPad Prism 5.0 (GraphPad Software Inc., San Diego, CA, USA). All data are expressed as the mean ± SEM from three independent experiments and were analyzed using one-way ANOVA with Tukey’s multiple comparison analysis. A *p* value < 0.05 was considered statistically significant.

## Figures and Tables

**Table 1:** Control, CYP1A1, and CYP1B1 siRNA sequences.

siRNA	Duplex Sequence		MW
Control	Sense	CCUCGUGCCGUUCCAUCAGGUAGUU	7487.7
	Antisense	CUACCUGAUGGAACGGCACGAGGUU	7636.9
CYP1A1	Sense	CUGGUAUUCUGGGUAAUCAUU	6316.1
	Antisense	UGAUUACCCAGAAUACCAGUU	6305.1
CYP1B1	Sense	GCAACUUCAGCAACUUCAUUU	6242.1
	Antisense	AUGAAGUUGCUGAAGUUGCUU	6379.2

**Table 2:** Gene primer sequences for RT-qPCR.

Gene	Sequence		Product Length (bp)
β-actin	Forward	AACTGGAACGGTGAAGGT	133
	Reverse	CCTGTAACAACGCATCTCATAT	
mTOR	Forward	GAAGAAGGTCACTGAGGAT	133
	Reverse	GGAGATGGAACGGAAGAA	
CYP1A1	Forward	CACAGCACAACAAGAGAC	128
	Reverse	TCAGGTAGGAACTCAGATG	
CYP1B1	Forward	TGGAGATGAGGTCAGTTG	181
	Reverse	AAGCACTTAGCACTTAGGA	
LC3	Forward	GAGCGAGTTGGTCAAGAT	188
	Reverse	CTCAGAAGCCGAAGGTTT	
P62	Forward	GCAGACCAAGAACTATGAC	123
	Reverse	CACAACTATGAGACAGAAGAG	
TFE3	Forward	TTGCTCCATCCTTTGTCT	159
	Forward	GTCTCATCCTCACTTCTGT	
LAMP2A	Forward	GCCATCTCCTACTACAACA	138
	Forward	ACTGAAGCAACCTTATCCT	
PLEKHM1	Forward	CCACAAACACATCATCTCAG	115
	Reverse	CAGGTAGCACTCCATCAG	

## Data Availability

The data of this study are available from the corresponding author (Jung-Suk Sung) upon reasonable request.

## Supplementary Materials

The following supporting information can be downloaded at: https://www.mdpi.com/article/10.3390/ijms25021324/s1.
