# Supplementary material for: Regulation of Benzo[a]pyrene-Induced Hepatic Lipid Accumulation through CYP1B1-Induced mTOR-Mediated Lipophagy
Source: Int J Mol Sci. 2024 Jan 22;25(2):1324. doi: 10.3390/ijms25021324 (PMC10816991; doi:10.3390/ijms25021324)
Supplement: Supplementary file 1 [file ijms-25-01324-s001.zip › ijms-2823776-supplementary.pdf]

Supplementary Table S1. Sequences of all siRNAs for CYP1A1 and CYP1B1 knockdown

siRNA		Duplex sequence		MW
Control		Sense	CCUCGUGCCGUUCCAUCAGGUAGUU	7487.7
		Antisense	CUACCUGAUGGAACGGCACGAGGUU	7636.9
CYP1A1	1	Sense	CUGGUAUUCUGGGUAAUCAUU	6316.1
		Antisense	UGAUUACCCAGAAUACCAGUU	6305.1
	2	Sense	GUAUCAGUGACCAAUGUCAUU	6322.1
		Antisense	UGACAUUGGUCACUGAUACUU	6299.1
	3	Sense	CCUUCAAGGACCUGAAUGAUU	5321.1
		Antisense	UCAUUCAGGUCCUUGAAGGUU	6315.1
	4	Sense	CACAACAAGAGACACAAGUUU	6374.2
		Antisense	ACUUGUGUCUCUUGUUGUGUU	6247
	5	Sense	GUGAGAAGGUGAUUAUCUUUU	6380.2
		Antisense	AAGAUAAUCACCUUCUCACUU	6226.1
CYP1B1	1	Sense	GCAUGAUGCGCAACUUCUUUU	6275.1
		Antisense	AAGAAGUUGCGCAUCAUGCUU	6361.2
	2	Sense	GCAACUUCAGCAACUUCAUUU	6242.1
		Antisense	AUGAAGUUGCUGAAGUUGCUU	6379.2
	3	Sense	GCGAAGAACUUUCUAAGAUUU	6346.2
		Antisense	AUCUUAGAAAGUUCUUCGCUU	6260.1
	4	Sense	CAGUUAUGGUCUAACCAUUUU	6260.1
		Antisense	AAUGGUUAGACCAUAACUGUU	6346.2
	5	Sense	CCAUUAAACCCAAGUCAUUUU	6226.1
		Antisense	AAUGACUUGGGUUUAAUGGUU	6380.2

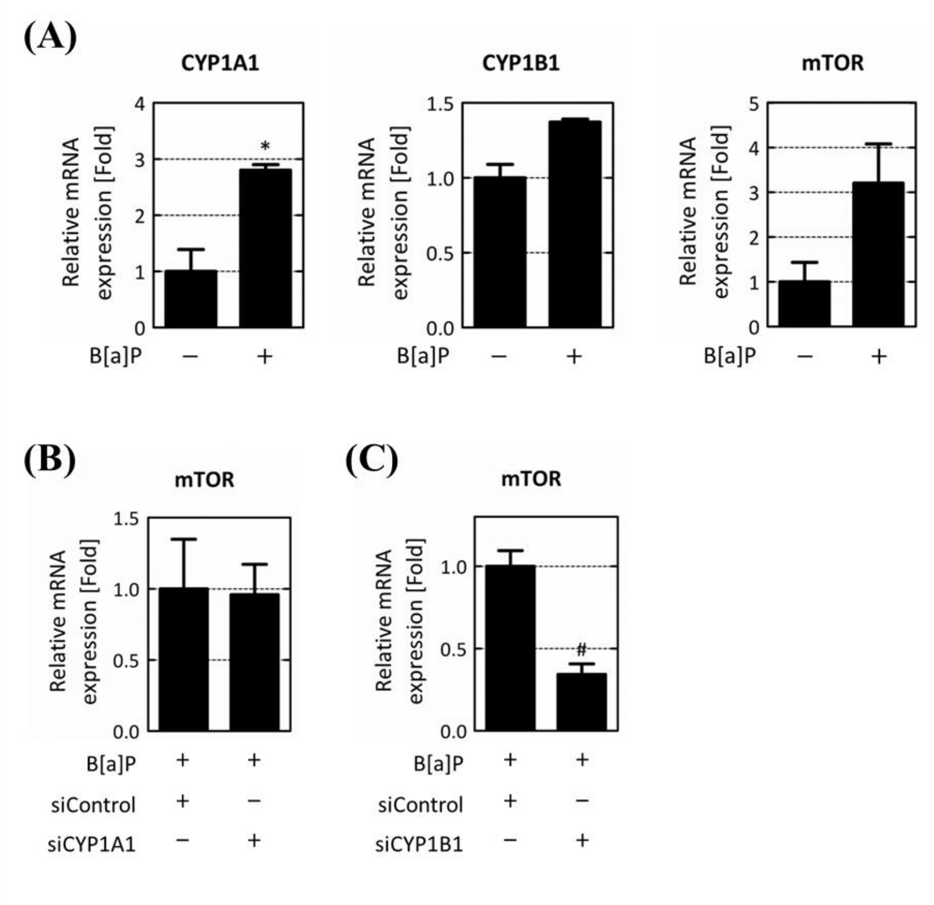

Supplementary Figure S1. mRNA levels of CYP1A1, CYP1B1, and mTOR after B[a]P treatment and knockdown of CYP1A1 and CYP1B1. (A) mRNA levels of CYP1A1, CYP1B1, and mTOR upon B[a]P treatment were analyzed by RT-qPCR. Relative mTOR mRNA expression levels were investigated by RT-qPCR following (B) CYP1A1 and (C) CYP1B1 knockdown, respectively. * $p < 0.05$, compared with the non-treated group; # $p < 0.05$, compared with the siControl group. B[a]P: benzo[a]pyrene (10 μM).

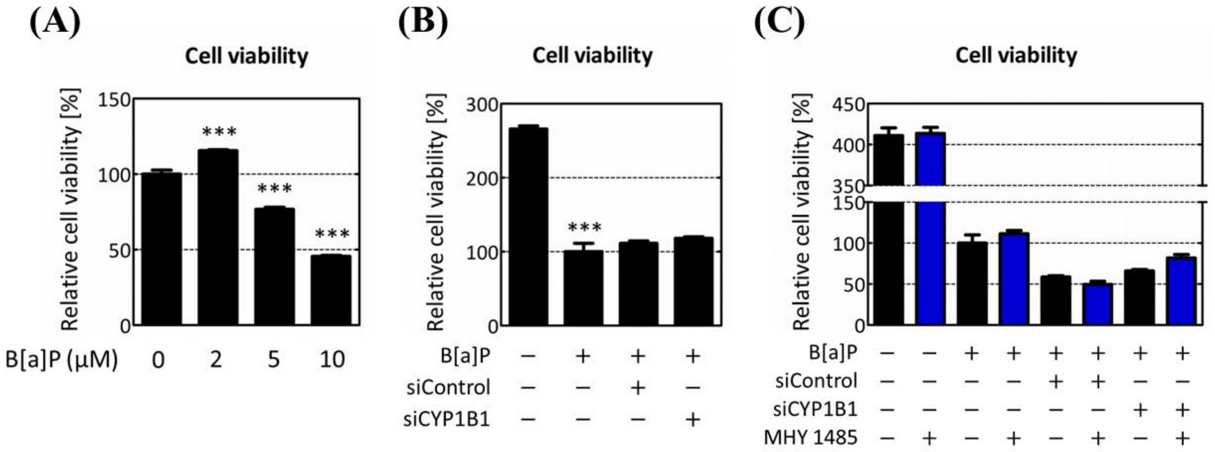

Supplementary Figure S2. Cell viability in response to treatment with B[a]P, siRNA, and MHY 1485. (A) Cell viability levels are represented in the graph over a range of B[a]P concentrations from 2 μM to 10 μM. (B) The cell viability levels according to CYP1B1 knockdown are shown in the graph. (C) Cell viability according to MHY 1485 was analyzed. *** $p < 0.001$, compared with the non-treated group. B[a]P: benzo[a]pyrene (10 μM); MHY 1485: mTOR activator (2 μM).

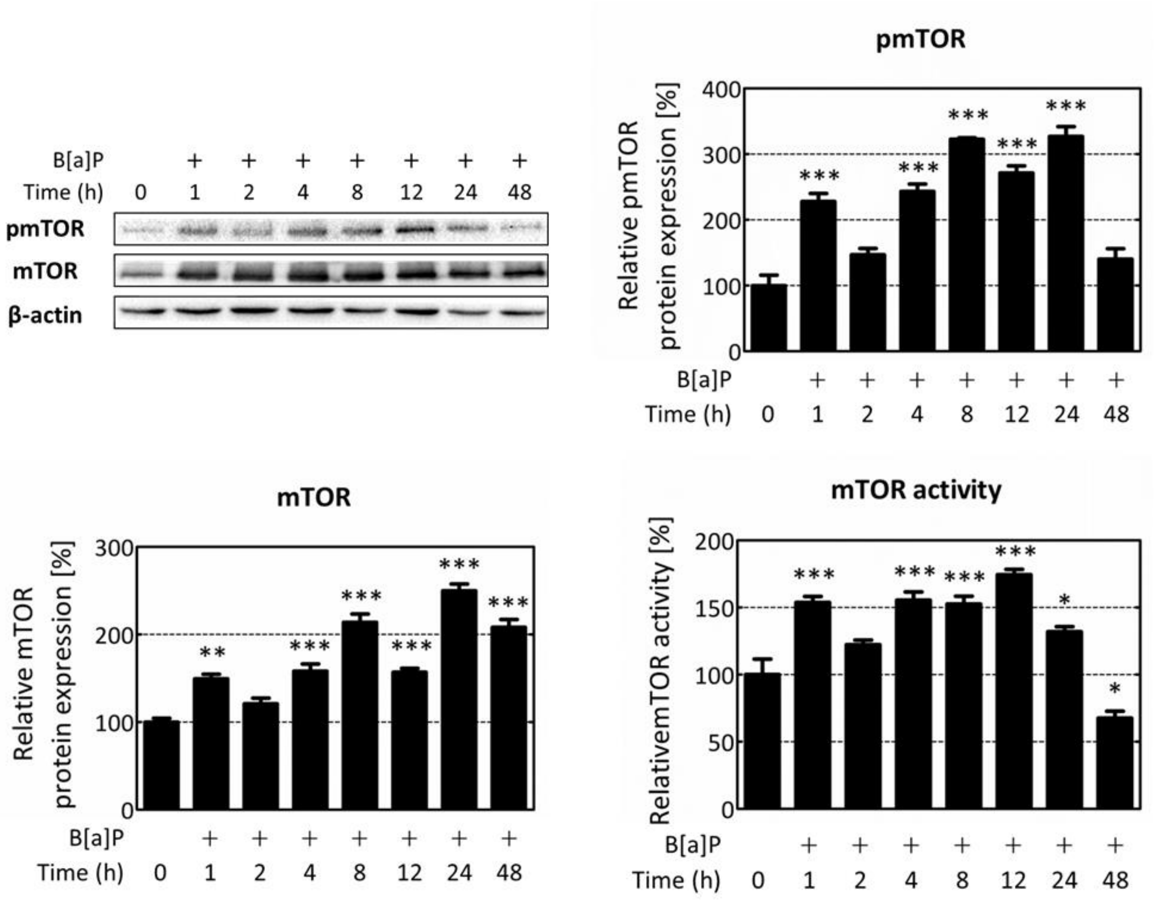

Supplementary Figure S3. Expression and activity of mTOR over the time course. pmTOR and mTOR protein expressions in response to B[a]P treatment were assessed by Western blot analysis to determine the optimal time period for treatment. Protein expression levels of pmTOR and mTOR were confirmed in the treatment range from 1 h to 48 h, and mTOR activity was confirmed accordingly. * $p < 0.05$, ** $p < 0.01$, *** $p < 0.001$, compared with the control group. B[a]P: benzo[a]pyrene (10 μM).

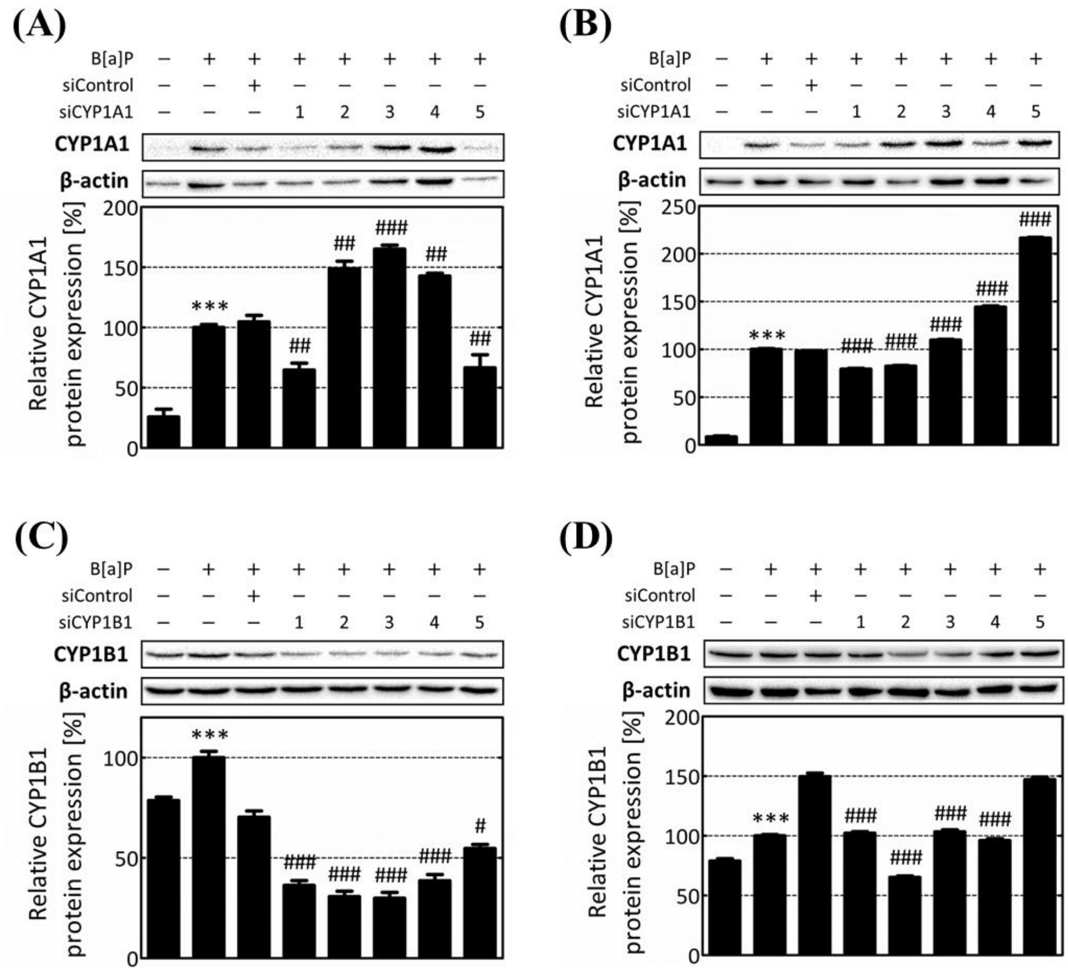

Supplementary Figure S4. Knockdown efficiency of CYP1A1 and CYP1B1 siRNAs. To select the optimal siRNA, the knockdown efficiencies for CYP1A1 and CYP1B1 siRNAs were confirmed. Knockdown efficiency of CYP1A1 siRNA over **(A)** 24 h and **(B)** 48 h. Knockdown efficiency of CYP1B1 siRNA over **(C)** 24 h and **(D)** 48 h. *** $p < 0.001$, compared with the non-treated group; # $p < 0.05$, ## $p < 0.01$, ### $p < 0.001$, compared with the siControl group. B[a]P: benzo[a]pyrene (10 μM).
